# Scrub Typhus-Associated Inflammatory Oligoarthritis Mimicking Tubercular Arthritis in an Endemic Setting

**DOI:** 10.7759/cureus.106318

**Published:** 2026-04-02

**Authors:** Amrit K C, Suyesh Poudel, Adarsha Mahaseth, Raj Kumar Thapa, Rajiv Sitaula, Prakash Shrestha

**Affiliations:** 1 Department of General Medicine, Dr. Iwamura Memorial Hospital, Bhaktapur, NPL; 2 Department of Medicine, Shree Birendra Hospital, Kathmandu, NPL; 3 Department of Medicine, Nepalese Army Institute of Health Sciences, Kathmandu, NPL; 4 Department of Medicine, Vayodha Hospital, Kathmandu, NPL

**Keywords:** culture-negative arthritis, differential diagnosis, doxycycline, febrile arthritis, oligoarthritis, orientia tsutsugamushi, scrub typhus, tropical infections, tubercular arthritis

## Abstract

Scrub typhus is a common cause of acute febrile illness in endemic regions; however, musculoskeletal involvement is usually limited to myalgia, and true inflammatory arthritis is rarely reported. We describe a 19-year-old previously healthy male military recruit who presented with a two-month history of progressive bilateral knee swelling and left ankle pain, accompanied by fever, weight loss, and lymphadenopathy. Clinical evaluation revealed inflammatory oligoarthritis with culture-negative synovial fluid. Given the chronicity of symptoms and systemic features, tubercular and septic arthritis were initially considered. Imaging showed preserved joint architecture, and synovial fluid analysis demonstrated inflammatory features without microbial growth. Investigations for tuberculosis, autoimmune disorders, and other tropical infections were negative. Serological testing was positive for scrub typhus immunoglobulin M using a rapid immunochromatographic assay. Following the initiation of doxycycline therapy, the patient showed rapid clinical improvement with resolution of fever and marked reduction in joint symptoms. This case highlights an uncommon presentation of scrub typhus-associated inflammatory oligoarthritis mimicking tubercular arthritis in endemic regions. Clinicians should consider scrub typhus in patients presenting with febrile oligoarthritis and culture-negative joint effusion, as early recognition and appropriate treatment can result in excellent outcomes.

## Introduction

Scrub typhus is an acute febrile zoonotic illness caused by* Orientia tsutsugamushi*, an obligate intracellular gram-negative bacterium transmitted to humans through the bite of infected larval trombiculid mites (chiggers) [1,2]. The disease is classically endemic within the "Tsutsugamushi Triangle," extending from northern Japan and far-eastern Russia through Southeast Asia to northern Australia [1-3]. More than one billion individuals are estimated to be at risk globally, with approximately one million cases occurring annually, making scrub typhus a significant yet frequently underrecognized cause of acute febrile illness in the Asia-Pacific region [2-4]. Although traditionally confined to this geographical area, emerging epidemiological evidence suggests expanding transmission beyond the classical triangle, with reported cases from the Middle East, Africa, and South America [2,3,5].

Clinically, scrub typhus demonstrates a broad spectrum of manifestations, ranging from mild, self-limiting febrile illness to severe multisystem disease [2,4,6]. Common features include fever, headache, myalgia, rash, lymphadenopathy, and occasionally an eschar at the site of the chigger bite [1,6]. Severe disease may involve acute respiratory distress syndrome, encephalitis, myocarditis, acute kidney injury, shock, and multi-organ dysfunction syndrome [1,6]. The underlying pathophysiology is characterized by endothelial infection and immune-mediated inflammation, resulting in systemic vasculitis [1,2]. Musculoskeletal involvement is typically limited to generalized myalgia, whereas true inflammatory arthritis with synovitis is rarely reported in the literature [6,7].

We report the case of a 19-year-old Nepali male military recruit who presented with persistent bilateral knee effusion and ankle pain associated with fever and significant weight loss, initially raising suspicion for tubercular and septic arthritis. In this demographic, sexually transmitted reactive arthritis is an important differential diagnosis; however, subsequent evaluation, including negative GeneXpert testing for *Mycobacterium tuberculosis* and absence of supportive clinical or laboratory evidence for alternative infectious etiologies, supported the exclusion of tubercular arthritis. Further investigations supported the diagnosis of scrub typhus-associated culture-negative inflammatory oligoarthritis. This case highlights an uncommon musculoskeletal manifestation and underscores the importance of considering scrub typhus in the differential diagnosis of febrile oligoarthritis in endemic regions, particularly where tuberculosis is highly prevalent.

## Case presentation

A 19-year-old previously healthy Nepali male military recruit presented with a two-month history of progressively worsening bilateral knee swelling accompanied by pain and discomfort in the left ankle. The swelling had an insidious onset and initially preceded the development of pain, gradually increasing in severity and ultimately resulting in significant difficulty with weight-bearing and ambulation. There was no history of trauma, excessive physical exertion, or prior joint disease. The patient denied any preceding gastrointestinal or genitourinary infection, conjunctivitis, mucocutaneous lesions, or previous similar episodes suggestive of classical reactive arthritis. There was no involvement of the small joints of the hands or feet.

Approximately one month prior to presentation, the patient reported significant unintentional weight loss of approximately 12 kg, associated with anorexia. Two weeks before admission, he developed intermittent fever characterized by evening rise, accompanied by chills, rigors, night sweats, and headache. There were no associated respiratory, gastrointestinal, or genitourinary symptoms. The patient denied recent travel outside Nepal and had no history of field deployment or jungle exposure. He also reported no known exposure to tuberculosis. The overall clinical course was therefore characterized by progressive oligoarticular joint involvement over two months, followed by constitutional symptoms for one month and the onset of febrile illness two weeks prior to presentation.

On admission, the patient was hemodynamically stable, with a blood pressure of 120/80 mmHg, a pulse rate of 74 beats per minute, and an oxygen saturation of 96% on room air. He was afebrile at presentation. General physical examination revealed bilateral inguinal lymphadenopathy, with tender, soft lymph nodes measuring approximately 2 cm in diameter. Local examination of the joints demonstrated mild warmth without overlying erythema. Musculoskeletal examination showed bilateral knee swelling with clinically evident joint effusion, along with painful and restricted active and passive movements. Tenderness was also elicited over the medial malleolus of the left ankle. No joint deformities were observed, and the small joints remained uninvolved. A meticulous dermatological examination did not reveal any eschar.

**Figure 1:**
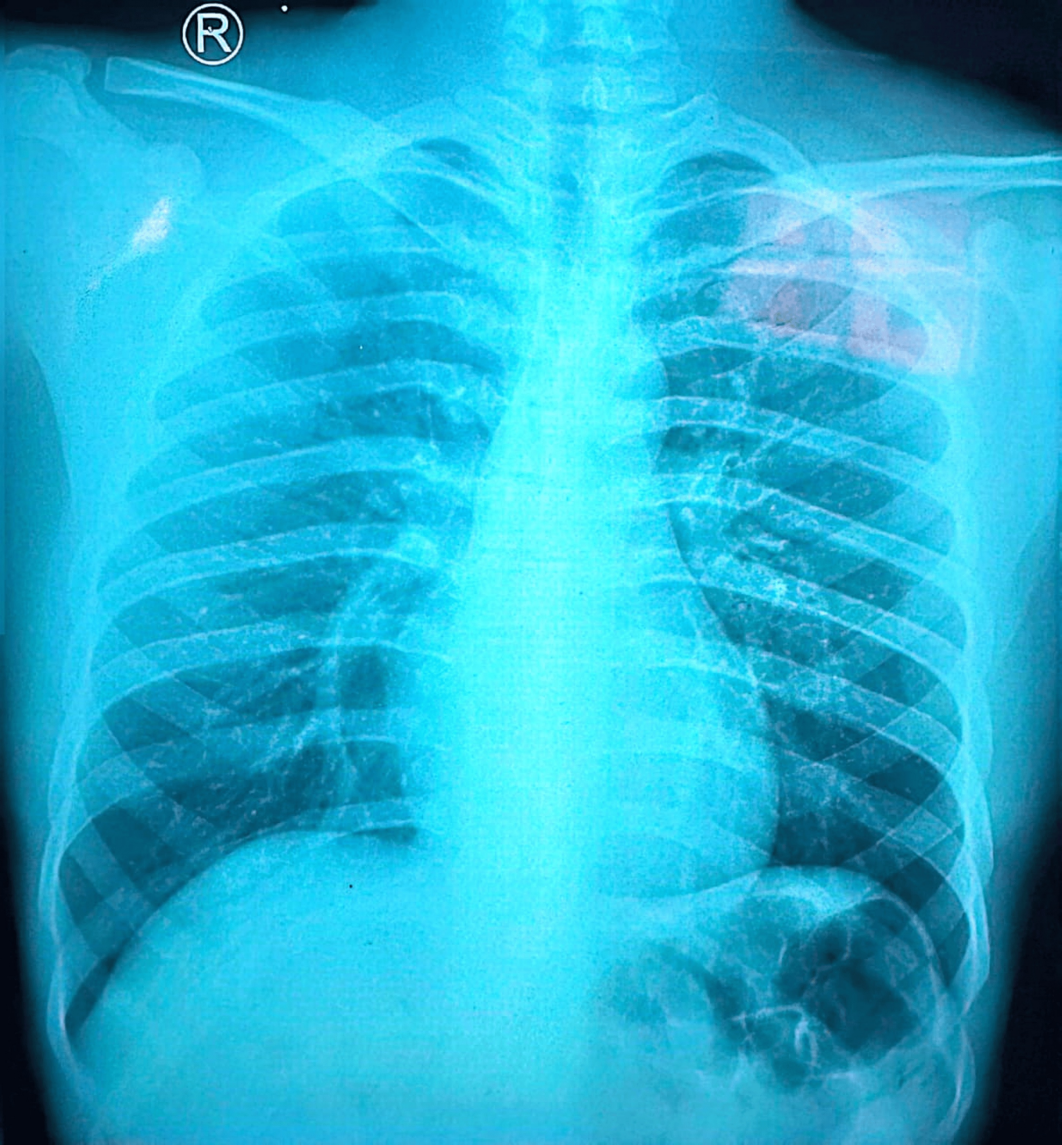
Chest radiograph (posteroanterior view) showing normal lung fields without consolidation, cavitation, or pleural effusion.

**Figure 2:**
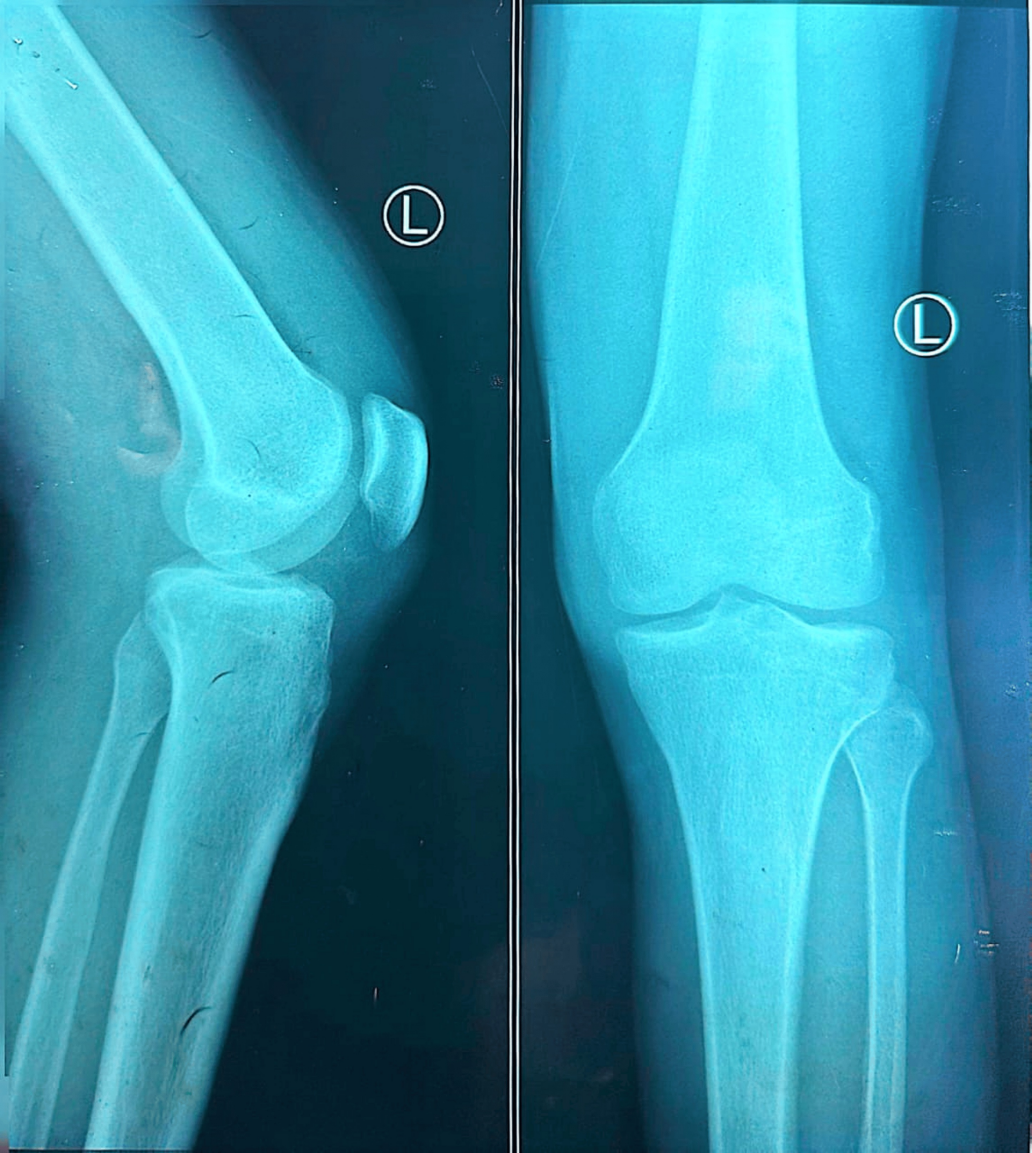
Plain radiograph of the left knee (anteroposterior and lateral views) showing preserved joint space and normal alignment without erosive changes.

**Figure 3:**
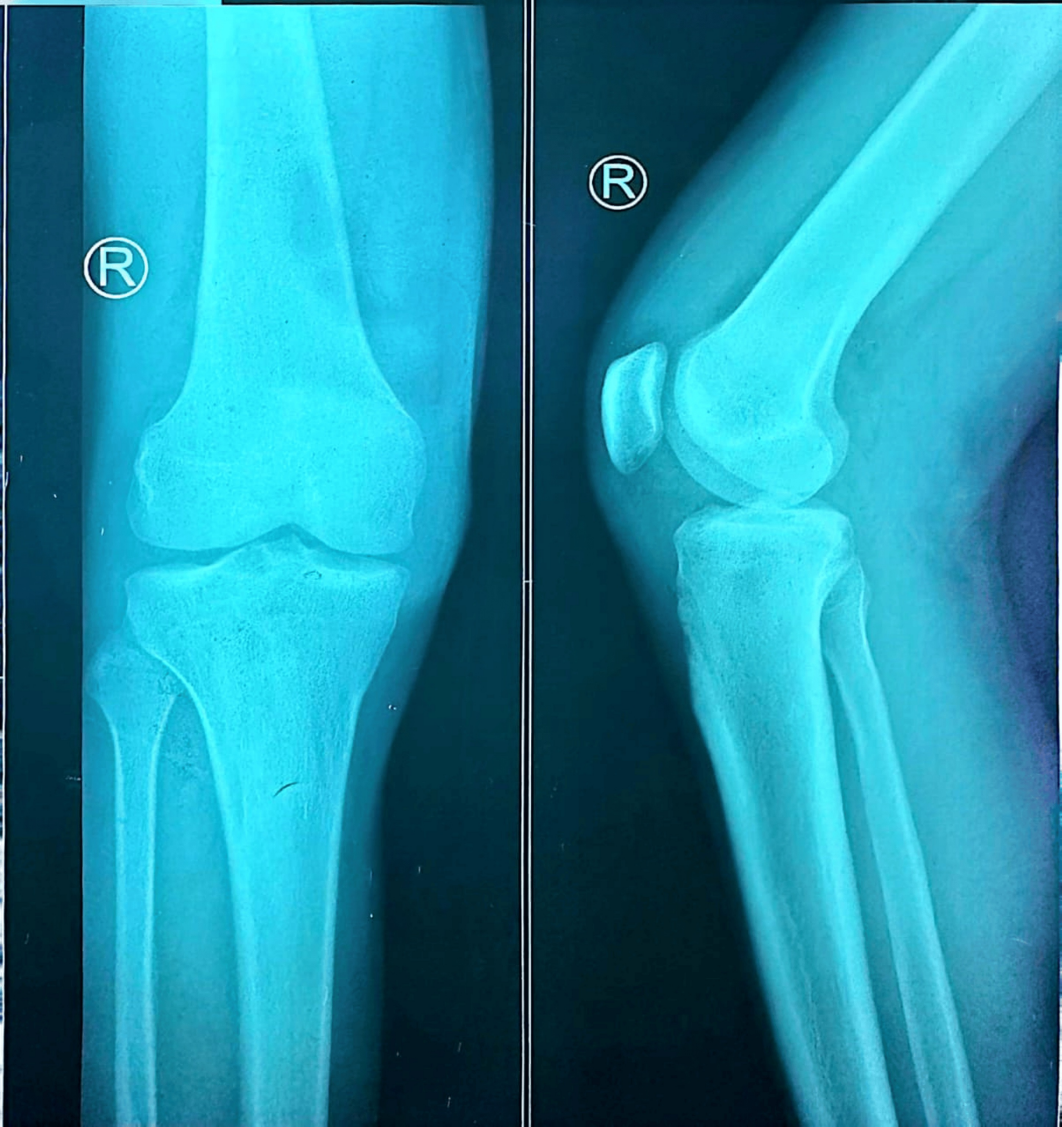
Plain radiograph of the right knee (anteroposterior and lateral views) demonstrating normal joint architecture without evidence of osteomyelitis or degenerative changes.

**Figure 4:**
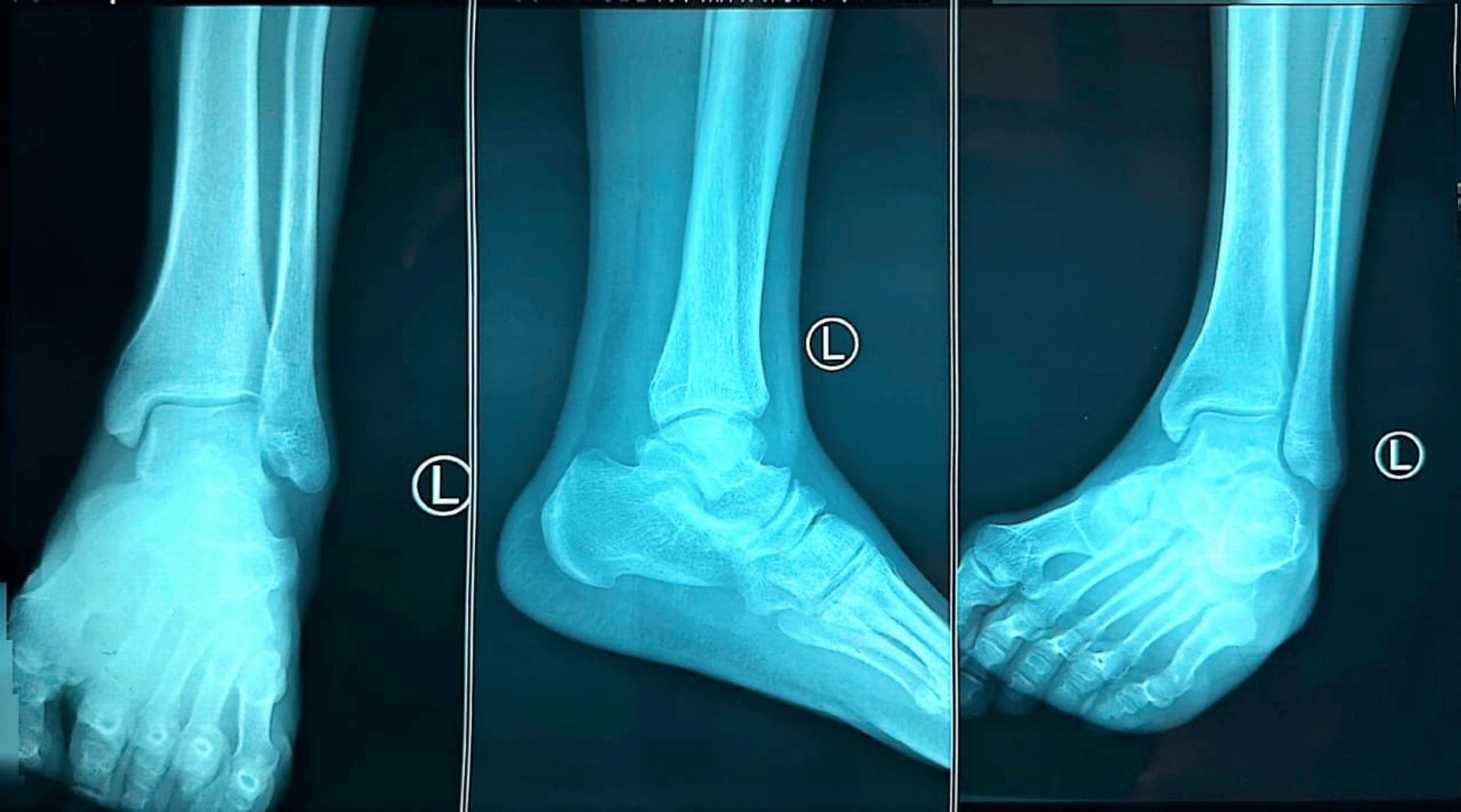
Plain radiograph of the left ankle (anteroposterior, lateral, and oblique views) showing preserved joint alignment and normal bony structures.

**Figure 5:**
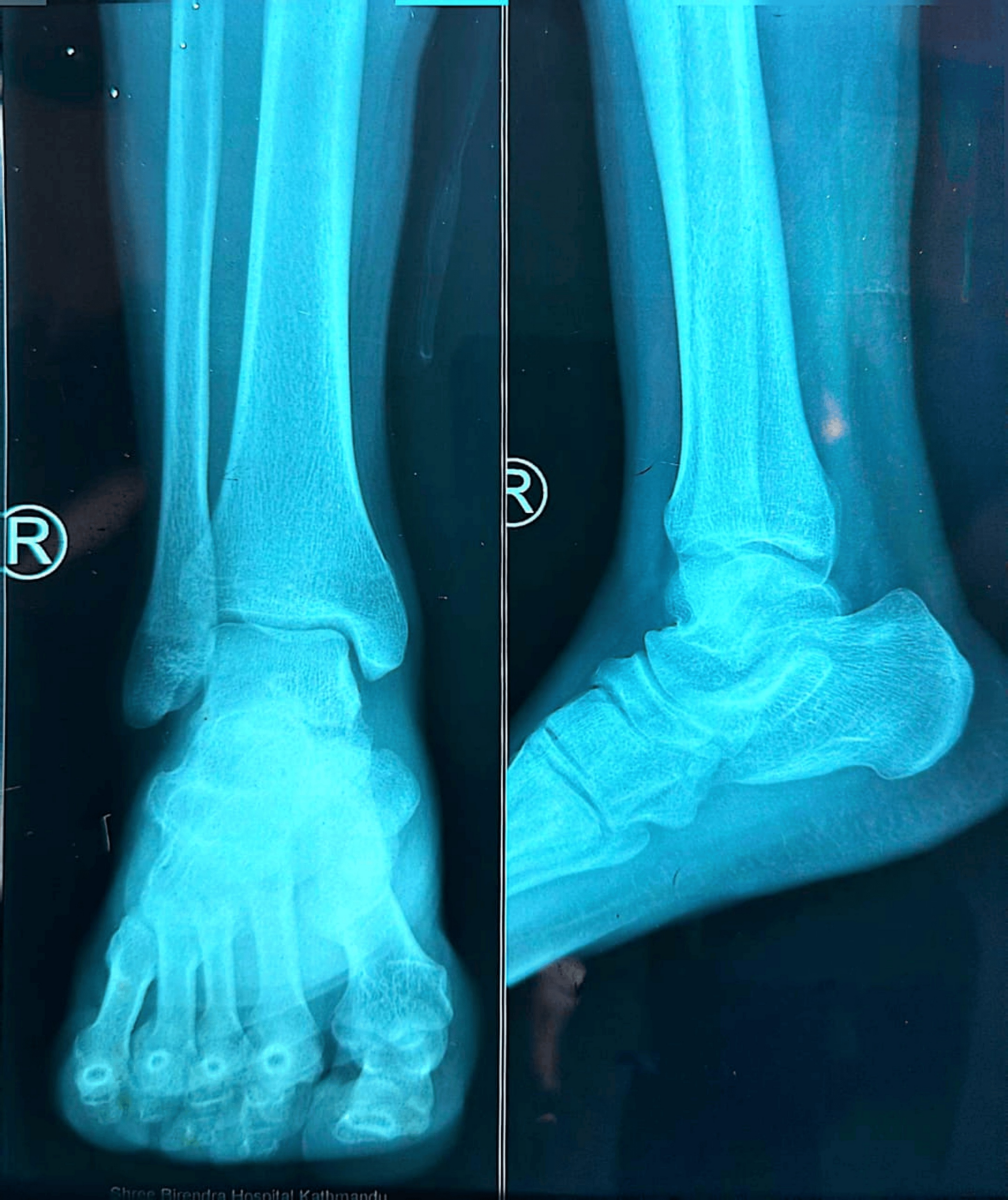
Plain radiograph of the right ankle (anteroposterior and lateral views) showing preserved joint alignment and normal bony structures.

Baseline imaging was performed to evaluate structural joint integrity and exclude destructive etiologies. A chest radiograph (Figure 1) demonstrated normal lung fields without consolidation, cavitation, or pleural effusion. Radiographs of both knees (Figure 2 and Figure 3) showed preserved joint spaces without erosions, osteolytic lesions, or periosteal reaction, with minimal degenerative changes suggestive of early osteoarthritis. Similarly, ankle radiographs (Figure 4 and Figure 5) revealed normal bony architecture and preserved joint alignment without evidence of osteomyelitis or degenerative changes. These findings supported the presence of an inflammatory joint process without structural bone involvement.

Given the chronic oligoarticular involvement in the context of systemic symptoms and the endemic setting, differential diagnoses included tubercular arthritis, septic arthritis, reactive arthritis, crystal-induced arthritis, and other tropical infectious etiologies. Tubercular arthritis was initially considered due to the chronicity of symptoms and significant weight loss.

**Figure 6:**
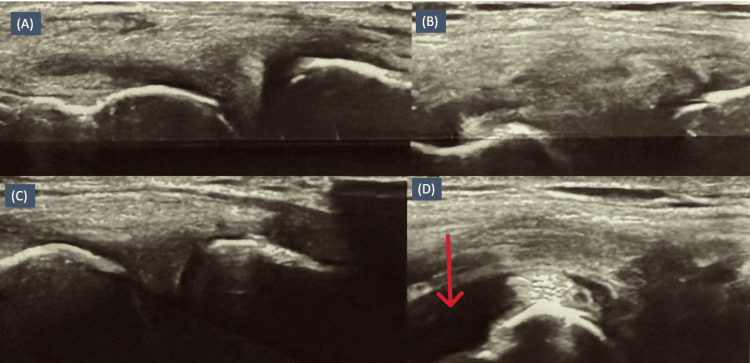
Ultrasonography of the left knee demonstrating mild joint effusion predominantly within the suprapatellar recess (arrow).

Ultrasonography of the left knee demonstrated mild (Grade I) joint effusion predominantly within the suprapatellar recess (Figure 6). Diagnostic arthrocentesis was performed under aseptic conditions. Synovial fluid analysis revealed inflammatory characteristics, with a total leukocyte count of 13,400 cells/mm³ and a differential count of 70% neutrophils and 30% lymphocytes. No crystals were identified. Gram staining did not reveal any organisms, and bacterial cultures showed no growth after 48 hours. The synovial fluid adenosine deaminase (ADA) level was 16 U/L, which was not suggestive of tubercular arthritis. These findings are summarized in Table 1. The moderate leukocyte count, culture-negative results, and lack of response to empirical antibiotic therapy argued against septic arthritis.

**Table 1:** Synovial fluid analysis. ADA: adenosine deaminase

Parameter	Finding
Appearance	Turbid
Total leukocyte count	13,400 cells/mm³
Differential	70% neutrophils, 30% lymphocytes
Crystals	Absent
Gram stain	Negative
Culture	No growth
ADA	16 U/L

Comprehensive laboratory evaluation demonstrated elevated inflammatory markers, with a C-reactive protein level of 13.78 mg/L and an erythrocyte sedimentation rate (ESR) of 48 mm/hour. Hematological parameters revealed mild anemia (hemoglobin 11.8 g/dL), while total leukocyte count and platelet count were within normal limits. Rheumatoid factor, Mantoux test, and human leukocyte antigen B27 (HLA-B27) were negative. These findings are summarized in Table 2.

**Table 2:** Laboratory investigations. CRP: C-reactive protein; ESR: erythrocyte sedimentation rate; HLA-B27: human leukocyte antigen B27

Parameter	Result	Reference range
Hemoglobin	11.8 g/dL	13-17 g/dL
Total leukocyte count	8,200/mm³	4,000-11,000/mm³
Neutrophils	67.6%	40-75%
Lymphocytes	20.9%	20-40%
Platelets	335,000/mm³	150,000-400,000/mm³
CRP	13.78 mg/L	<5 mg/L
ESR	48 mm/hour	<20 mm/hour
Serum uric acid	4.52 mg/dL	3.4-7.0 mg/dL
Rheumatoid factor	Negative	Negative
Mantoux test	Negative	Negative
HLA-B27	Negative	Negative

Liver function tests demonstrated mild transaminitis, with elevated aspartate aminotransferase (AST) and alanine aminotransferase (ALT) levels and otherwise preserved hepatic parameters, consistent with mild hepatic involvement. These findings are summarized in Table 3.

**Table 3:** Liver function tests. AST: aspartate aminotransferase; ALT: alanine aminotransferase; ALP: alkaline phosphatase

Parameter	Result	Reference range
AST	65 U/L	10-40 U/L
ALT	52 U/L	7-56 U/L
ALP	116 U/L	44-147 U/L
Total bilirubin	1.1 mg/dL	0.2-1.2 mg/dL
Albumin	3.7 g/dL	3.5-5.0 g/dL

Additional laboratory evaluation was performed to exclude other infectious and differential diagnoses, including sexually transmitted infections and viral hepatitis. Serological testing for scrub typhus was performed using a rapid immunochromatographic immunoglobulin M (IgM) assay, which yielded a reactive result. All results were negative, as summarized in Table 4.

**Table 4:** Exclusion of competing infectious and differential diagnoses. NS1: non-structural protein 1; IgM: immunoglobulin M; IgG: immunoglobulin G; HIV: human immunodeficiency virus; VDRL: venereal disease research laboratory; RPR: rapid plasma reagin; HBsAg: hepatitis B surface antigen; HCV: hepatitis C virus

Test	Result	Interpretation
Dengue (NS1, IgM/IgG)	Negative	Excluded
Malaria	Negative	Excluded
Leptospirosis	Negative	Excluded
Brucellosis	Negative	Excluded
Kala-azar	Negative	Excluded
HIV	Non-reactive	Immunosuppression unlikely
VDRL/RPR	Non-reactive	STI unlikely
HBsAg	Non-reactive	Hepatitis B excluded
Anti-HCV	Non-reactive	Hepatitis C excluded
Rheumatoid factor	Negative	Autoimmune unlikely
Uric acid	Normal	Gout unlikely

Tubercular arthritis remained an important consideration given the clinical context. However, the absence of radiographic destruction, low synovial fluid ADA levels, a non-reactive Mantoux test, and culture-negative synovial fluid findings argued against this diagnosis. GeneXpert testing for *Mycobacterium tuberculosis* was also negative. Septic arthritis was considered unlikely due to negative gram staining and culture results, along with a lack of response to empirical antibiotic therapy.

A comparative diagnostic framework is provided in Table 5, highlighting distinguishing features among the major differentials.

**Table 5:** Diagnostic differentiation of major conditions. ADA: adenosine deaminase; MTB: *Mycobacterium tuberculosis* This table is an original creation of the authors.

Feature	Tubercular arthritis	Septic arthritis	Scrub typhus-associated arthritis (this case)
Onset	Chronic	Acute	Subacute
Fever	Low-grade	High	Present
Weight loss	Common	Rare	Present
Synovial fluid	Lymphocytic	Neutrophilic (high)	Culture-negative inflammatory
ADA	Elevated	Normal	Low
Culture	May detect MTB	Often positive	Negative
GeneXpert	May be positive	Not applicable	Negative
Radiology	Destructive	Initially normal progressing to destructive	Normal
Response to therapy	Requires anti-tubercular therapy	Antibiotics	Rapid response to doxycycline

The overall clinical picture, supported by serological evidence, including a reactive rapid immunochromatographic IgM assay for scrub typhus, systematic exclusion of competing diagnoses, and subsequent therapeutic response, was most consistent with scrub typhus-associated culture-negative inflammatory oligoarthritis.

The patient was initially treated empirically with intravenous flucloxacillin for presumed septic arthritis; however, no clinical improvement was observed. Following the recognition of scrub typhus as the most likely etiology, oral doxycycline (100 mg twice daily) was initiated.

Following the initiation of doxycycline therapy, the patient demonstrated rapid clinical improvement. Fever subsided within 48-72 hours, lymphadenopathy regressed, and joint swelling progressively decreased. By day 6, the patient was able to ambulate with minimal discomfort, and by day 7, only mild residual ankle tenderness persisted.

The patient was discharged on day 7 of doxycycline therapy with advice for follow-up. At two weeks, he reported complete resolution of fever and significant improvement in joint symptoms. At six weeks, there was complete resolution of knee effusion. At three months of follow-up, the patient remained asymptomatic, with no recurrence.

## Discussion

This case highlights an uncommon presentation of scrub typhus manifesting as culture-negative inflammatory oligoarthritis, initially mimicking tubercular or septic arthritis. Although scrub typhus is a well-recognized cause of acute undifferentiated febrile illness in endemic regions, joint-predominant presentations remain rare and may lead to significant diagnostic uncertainty [3,7]. In the present case, persistent knee effusion with systemic symptoms and culture-negative synovial fluid raised suspicion for alternative infectious or inflammatory etiologies before serological testing subsequently supported the diagnosis of scrub typhus.

Musculoskeletal manifestations of scrub typhus are most commonly limited to generalized myalgia, whereas true inflammatory arthritis is infrequently reported [6,7]. Nevertheless, emerging case reports have expanded the recognized clinical spectrum of this infection. Khatri et al. described a young Nepali patient presenting with HLA-B27-negative reactive monoarthritis of the hip associated with scrub typhus, which resolved rapidly following doxycycline therapy [8]. Similarly, Handattu et al. reported a pediatric case of severe hip monoarthritis initially suspected to be septic arthritis but later attributed to scrub typhus after serological confirmation [9]. Polyarticular inflammatory involvement has also been described in both pediatric and adult populations [10]. In another report, Yamanaka et al. documented polymerase chain reaction (PCR)-confirmed scrub typhus associated with reactive shoulder monoarthritis, further supporting immune-mediated joint involvement [11]. Collectively, these observations indicate that although uncommon, inflammatory or reactive arthritis represents a plausible manifestation within the clinical spectrum of scrub typhus [8-11].

The underlying mechanism of joint involvement in scrub typhus is believed to be predominantly immune-mediated rather than due to direct bacterial invasion. Infection with *Orientia tsutsugamushi *leads to endothelial injury and perivascular inflammation, resulting in systemic vasculitis [1,2]. Cytokine-mediated immune activation has also been implicated in disease severity and multisystem involvement [12]. Experimental studies have demonstrated early infection of dendritic cells and monocytes within eschars, suggesting a pathway for immune activation and systemic dissemination [13]. The presence of culture-negative inflammatory synovial fluid, as observed in previously reported cases and in our patient, supports a reactive or immune-mediated mechanism, although direct infection cannot be completely excluded without molecular testing [8-11].

Recognition of such atypical presentations is clinically important. In endemic settings, scrub typhus should be considered in the differential diagnosis of febrile arthritis, particularly when synovial cultures show no growth on routine media and systemic features such as lymphadenopathy or constitutional symptoms are present. The negative GeneXpert result in this case further reduced the likelihood of tubercular arthritis, although the sensitivity of molecular testing in paucibacillary osteoarticular disease remains limited. Failure to recognize this association may result in unnecessary invasive procedures or inappropriate prolonged therapy. Previous reports have demonstrated rapid clinical improvement following the initiation of doxycycline, with resolution of both systemic and joint symptoms within a few days [1,6-11]. A similar therapeutic response was observed in our patient, which supports the diagnosis in the appropriate clinical context but does not establish definitive causality, given the broad antimicrobial spectrum of doxycycline.

This report has certain limitations. Although the diagnosis was supported by positive IgM serology, molecular confirmation using PCR was not performed. Tubercular arthritis could not be definitively excluded, as synovial biopsy and mycobacterial culture were not undertaken. Furthermore, synovial fluid analysis did not include molecular testing for *Orientia tsutsugamushi,* and advanced immunological characterization was not performed. Targeted testing for other doxycycline-responsive pathogens, such as *Chlamydia*, *Mycoplasma*, and *Coxiella*, was not performed; therefore, alternative or co-existing infections cannot be completely excluded. As a single case report, the findings may not be generalizable, and the duration of follow-up was limited.

## Conclusions

This case highlights an uncommon presentation of scrub typhus manifesting as culture-negative inflammatory oligoarthritis, closely mimicking tubercular and septic arthritis in endemic settings. The presence of persistent joint effusion, systemic features, and culture-negative synovial fluid can create significant diagnostic ambiguity and may lead to unnecessary investigations or inappropriate empirical therapy. In the present case, the diagnosis was supported by serological evidence and the systematic exclusion of alternative etiologies. The observed clinical improvement following doxycycline therapy further supports the diagnosis in the appropriate clinical context, although it does not establish definitive causality. Clinicians practicing in endemic regions should maintain a high index of suspicion for scrub typhus in patients presenting with febrile oligoarthritis, particularly when synovial cultures show no growth on routine media and conventional evaluations are inconclusive. Early recognition and timely initiation of appropriate therapy are essential, as they can result in rapid clinical recovery while preventing misdiagnosis, invasive procedures, and prolonged or inappropriate treatment.
